# DNA Structure Modulates the Oligomerization Properties of the AAV Initiator Protein Rep68

**DOI:** 10.1371/journal.ppat.1000513

**Published:** 2009-07-10

**Authors:** Jorge Mansilla-Soto, Miran Yoon-Robarts, William J. Rice, Shailee Arya, Carlos R. Escalante, R. Michael Linden

**Affiliations:** 1 Department of Gene & Cell Medicine, Mount Sinai School of Medicine, New York, New York, United States of America; 2 New York Structural Biology Center, New York, New York, United States of America; 3 Department of Structural and Chemical Biology, Mount Sinai School of Medicine, New York, New York, United States of America; 4 Department of Physiology and Biophysics, Virginia Commonwealth University School of Medicine, Richmond, Virginia, United States of America; 5 Department of Infectious Diseases, King's College London School of Medicine, Guy's, King's College and St. Thomas' Hospitals, London, United Kingdom; Yale School of Medicine, United States of America

## Abstract

Rep68 is a multifunctional protein of the adeno-associated virus (AAV), a parvovirus that is mostly known for its promise as a gene therapy vector. In addition to its role as initiator in viral DNA replication, Rep68 is essential for site-specific integration of the AAV genome into human chromosome 19. Rep68 is a member of the superfamily 3 (SF3) helicases, along with the well-studied initiator proteins simian virus 40 large T antigen (SV40-LTag) and bovine papillomavirus (BPV) E1. Structurally, SF3 helicases share two domains, a DNA origin interaction domain (OID) and an AAA^+^ motor domain. The AAA^+^ motor domain is also a structural feature of cellular initiators and it functions as a platform for initiator oligomerization. Here, we studied Rep68 oligomerization *in vitro* in the presence of different DNA substrates using a variety of biophysical techniques and cryo-EM. We found that a dsDNA region of the AAV origin promotes the formation of a complex containing five Rep68 subunits. Interestingly, non-specific ssDNA promotes the formation of a double-ring Rep68, a known structure formed by the LTag and E1 initiator proteins. The Rep68 ring symmetry is 8-fold, thus differing from the hexameric rings formed by the other SF3 helicases. However, similiar to LTag and E1, Rep68 rings are oriented head-to-head, suggesting that DNA unwinding by the complex proceeds bidirectionally. This novel Rep68 quaternary structure requires both the DNA binding and AAA^+^ domains, indicating cooperativity between these regions during oligomerization *in vitro*. Our study clearly demonstrates that Rep68 can oligomerize through two distinct oligomerization pathways, which depend on both the DNA structure and cooperativity of Rep68 domains. These findings provide insight into the dynamics and oligomeric adaptability of Rep68 and serve as a step towards understanding the role of this multifunctional protein during AAV DNA replication and site-specific integration.

## Author Summary

Adeno-associated virus (AAV) is a parvovirus with a linear single-stranded DNA genome. Thus far, it is the only eukaryotic virus known to integrate its genome in human cells in a specific region of chromosome 19. Because no pathologies have been associated with AAV, there is great interest in using AAV as a vector for gene therapy. The genetic information of AAV encodes for both the structural Capsid proteins and the Rep proteins. We have studied a protein called Rep68, which is essential for both AAV genome replication and site-specific integration in chromosome 19, and found that it forms distinct structures in the presence of different DNA structures. Of particular interest is the formation of a Rep68 structure composed of two opposite rings, which resemble the structures formed by the large T antigen and E1 viral proteins of the tumor-inducing Simian virus 40 (SV40) and papilloma viruses, respectively. The double-ring structure of these viral proteins is essential for viral DNA replication, which suggests that AAV has evolved a similar mechanism of DNA replication that relies on a double-ring Rep68. Moreover, Rep68 encounters different DNA structures during viral genome replication, and our results show how Rep68 can adapt to these changes.

## Introduction

Adeno-associated virus (AAV) is a non-pathogenic human parvovirus that has evolved a unique mechanism of persistence in human cells by integrating its genome site-specifically into a defined locus of human chromosome 19 [1]. The single-stranded AAV DNA genome contains two open reading frames (ORFs), REP and CAP that are flanked by inverted terminals repeats (ITRs). The non-structural proteins of the REP ORF mediate AAV DNA replication, integration, transcriptional regulation and packaging of the AAV genome into preformed empty capsids. The REP ORF encodes four Rep isoforms, Rep40, Rep52, Rep68, and Rep78 [2]. All Rep isoforms share a central AAA^+^ domain, which has ATPase and DNA helicase activities. Rep68 and Rep78 also contain the OID, which binds and nicks the ITR structure. Furthermore, Rep52 and Rep78 share a putative zinc-finger domain, which has been implicated in interacting with diverse cellular factors. Despite the apparent redundancy of functional domains, the biological functions of the small and the large Reps differ. Rep40 and Rep52 support efficient packaging of AAV DNA into AAV capsids [3],[4]. Rep68 and Rep78, on the other hand, are essential for AAV DNA replication [5]–[7] as well as site-specific integration of AAV DNA into human chromosome 19 at the AAVS1 locus [8].

The functional versatility shown by the AAV Rep proteins is in large part due to the presence of the AAA^+^ motor domain that structurally defines members of helicase superfamily 3 (SF3) [9]. SF3 helicases are multifunctional proteins only found in small DNA and RNA viruses such as simian virus 40 (SV40), bovine papillomavirus (BPV), and AAV. In addition to their DNA unwinding activity, LTag, E1, and Rep68/78 helicases act as initiators of DNA replication on their respective viral origins [10]. This function is facilitated by the presence of the OID, which is positioned at the amino-terminus of the AAA^+^ motor domain. Once bound to the origin of replication, DNA melting of Ori sequences promotes the formation of an active helicase oligomer, which in the case of SV40 LTag and BPV E1 is double-hexameric ring. To date, the oligomeric nature of the AAV Rep initiation complex remains inconclusive. The oligomeric character of the large Rep68/Rep78 is still under debate due to their tendency to aggregate at low ionic strength conditions [11]. Several studies have suggested that Rep68/Rep78 form hexameric rings upon binding AAV origin, however the supporting evidence is not entirely conclusive [11]–[13]. Moreover, in contrast to the corresponding AAA^+^ motor domains from SV40 LTag and BPV E1, the minimal AAA^+^ domain represented by Rep40 is monomeric [14],[15]. Thus AAV Rep proteins stand apart from the other SF3 family members, as they appear to have evolved an additional requirement for cooperative involvement of both the OID and the AAA^+^ domain for oligomerization. It is tempting to speculate that this step is further regulated by ATP binding as well as by the nature of the various DNA targets these multifunctional proteins encounter during the replication and integration process. Accordingly, we hypothesized that Rep68/Rep78 assembles into different complexes depending on the nature of the DNA substrates.

In order to address these questions we carried out a series of biochemical, biophysical, and structural studies using Cryo-EM and protein modeling in order to characterize the oligomeric nature of Rep68 in the absence and presence of different DNA substrates. Our analyses show that Rep68 assembles into a ring-shaped double octamer in the presence of ssDNA or ssDNA-dsDNA heteroduplex substrates. In contrast and consistent with previous suggestions, Rep68 assembles into a smaller complex in presence of RBS containing dsDNA. However, our analyses suggest this complex to be pentameric rather than hexameric as was proposed previously [11]–[13]. These results indicate a dynamic process during which Rep68 adopts different quaternary structures at distinct steps throughout the AAV DNA replication reaction.

## Results

### Rep68 forms different oligomeric complexes upon binding RBS dsDNA or ssDNA

Rep68 has two functional domains with independent DNA binding properties that are used at different stages of the viral life cycle: the OID binds the RBS double-stranded DNA specifically, while the AAA^+^ domain binds ssDNA or ss-dsDNA junctions non-specifically to perform the unwinding of DNA. We hypothesized that different oligomeric Rep68-DNA complexes are formed to carry out these diverse reactions.

**Figure 1:**
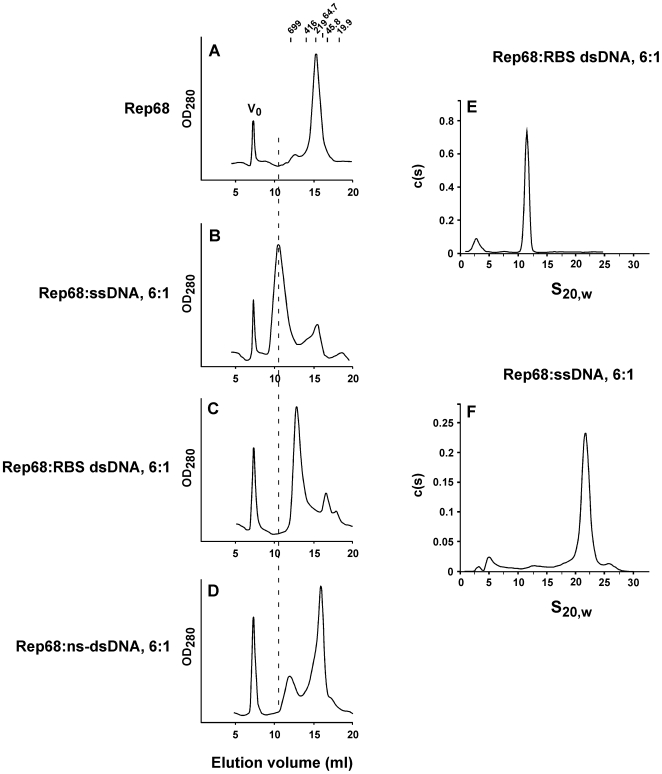
Rep68 oligomerizes in the presence of both sequence-specific and non-specific DNA. Rep68 (16.6 µM: 1 mg/ml) was incubated in the absence (A) or presence of 2.8 µM ssDNA (B), 2.8 µM RBS dsDNA (C), or 2.8 µM non-specific dsDNA (D). Fifty µL of sample was chromatographed on a Superose 6 10/300 GL column with a flow rate of 0.5 mL/min. Protein elution was followed by UV detection at 280 nm. X axis represents the elution volume (V_e_, in ml). Molecular weight standards and V_0_ position are shown in panel A. Sedimentation velocity data was obtained for the Rep68-RBS (E) and the Rep68-ssDNA (F) complexes at 20°C in buffer A, with Rep68 and DNA concentrations at 1 mg/ml and 2.8 µM, respectively.

We first used size exclusion chromatography in order to investigate the *in vitro* oligomerization properties of Rep68 after binding either a 26-mer RBS dsDNA sequence or a 25-mer poly-dT oligonucleotide. Both complexes were analyzed on a Superose-6 column that was calibrated with proteins of known Stokes radii. As expected, the two complexes elute at different times: the Rep68-RBS complex elutes with an apparent molecular weight of ∼578 kDa (Figure 1C), while the Rep68-ssDNA complex elutes earlier, with an apparent molecular weight of ∼2.3 MDa (Figure 1B). The calculated Stokes radius indicates that the Rep68-ssDNA complex is roughly twice as large as the Rep68-RBS complex (106 Å and 73.9 Å respectively; Figure S1A and B). In the presence of non-specific dsDNA substrates (Figure 1D), Rep68 did not efficiently oligomerize, although a slight difference in the elution profile can be observed when this complex is compared to apo Rep68 (Figure 1A).

Purified Rep68-RBS and Rep68-ssDNA complexes were further analyzed by small-angle X-ray scattering (SAXS), and the radii of gyration were determined to correspond to 81.65+/−2.34 Å and 154.895+/−1.327 Å, respectively (Figure S1C). These data are in agreement with the gel filtration results. Taken together, these results show that Rep68 can form different oligomers depending on the DNA substrate.

### RBS dsDNA promotes the formation of a pentameric Rep68 complex

In order to further examine the molecular weights of both Rep68 complexes, sedimentation velocity experiments were performed. Figure 1E shows that Rep68-RBS complex sediments with a coefficient S_20,w_ of 11.5S (Figure 1E), while Rep68-ssDNA complex sediments faster, with a sedimentation coefficient S_20,w_ of 21.9S (Figure 1F). A MW of ∼318 kDa was calculated for the Rep68-RBS complex. In contrast, for the Rep68-ssDNA complex a MW of ∼1 MDa was determined (Table 1). We further analyzed the Rep68-RBS complex using sedimentation equilibrium (SE) ultracentrifugation using two different concentrations at three increasing speeds. The complex was first purified by gel filtration and concentrated before SE. Global fitting yielded a molecular weight of ∼311 kDa for the complex at low complex concentrations, and ∼324 kDa when the concentration was 3-fold higher. Both values are in agreement with the value calculated from sedimentation velocity experiments (∼318 kDa). Taking into account that the theoretical MWs for pentameric and hexameric Rep68-RBS complexes with one DNA molecule are 321.3 kDa and 382.2 kDa, respectively, our data indicate that Rep68 assembles on the RBS DNA rather as a pentamer than the previously proposed hexamer [11]–[13]. The observed discrepancy with the molecular weights determined by gel filtration are likely due to the non-spherical nature of both complexes as suggested by their high frictional coefficient ratios f/f0 (1.79 and 1.83 for Rep68-RBS and Rep68-ssDNA complexes, respectively).

**Table 1:** Sedimentation coefficient and estimated molecular weights of the Rep68-RBA and Rep68-ssDNA complexes.

Sample	S_20,w_	SV - MW_est._ (Da)	SE - MW_est._ (Da)
Rep68 (1 mg/ml)/2.8 µM RBS DNA	11.5	318,789+/−39,876	
Rep68 (1 mg/ml)/2.8 µM ssDNA	21.9	908,065+/−97,410	
Purified Rep68-RBS DNA (OD_260_ = 0.25)			311,338+/−2420
Purified Rep68-RBS DNA (OD_260_ = 0.75)			324,277+/−1117

Sedimentation velocity (SV) and equilibrium (SE) experiments were performed as described in Materials and Methods. S_20,W_ coefficients and the estimated MWs from SV data were obtained with Sedfit. Molecular weight estimation of the Rep68-RBS complex from the SE data was calculated using HeteroAnalysis.

### Oligomerization of Rep68 on ssDNA requires cooperativity between the OID and AAA^+^ domains

**Figure 2:**
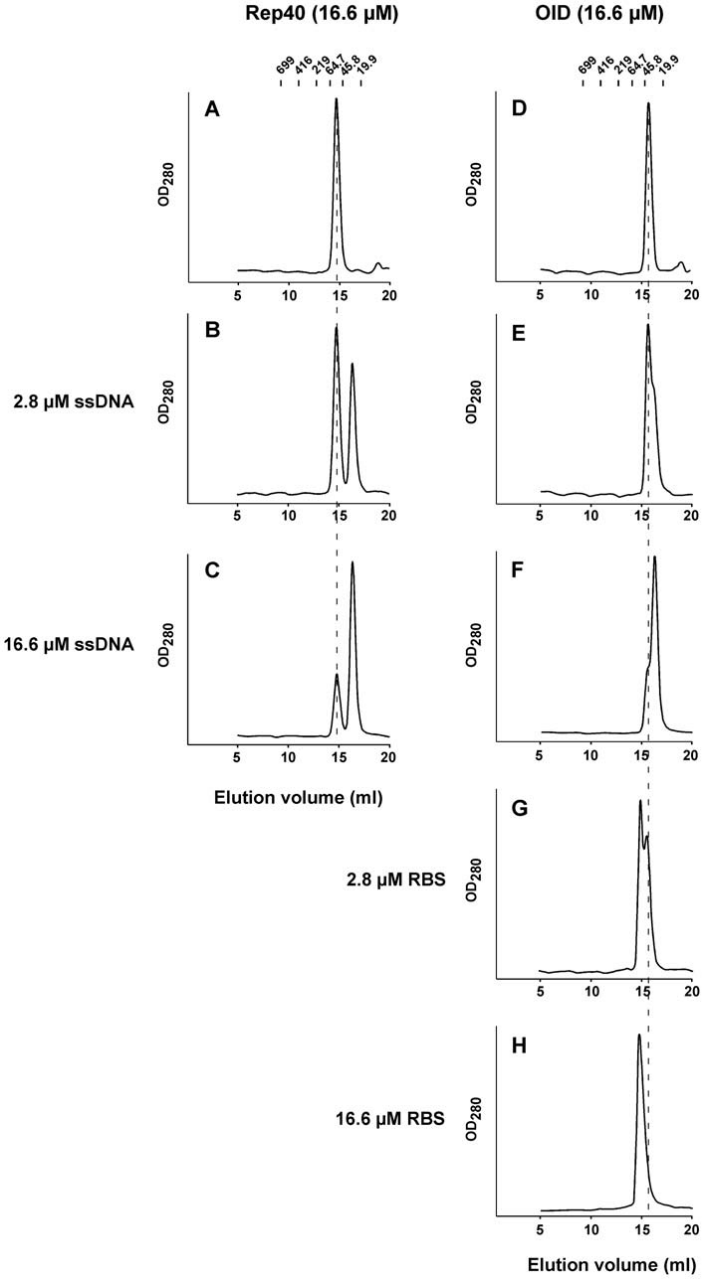
Origin interaction domain (OID) is required but not sufficient for ssDNA-dependent Rep68. Rep40 and OID proteins (16.6 µM) were incubated in the absence (A and D) or presence of 2.8 (B and E) or 16.6 µM ssDNA (C and F). OID was also incubated in the presence of 2.8 (G) or 16.6 µM RBS dsDNA (H). Fifty µL of sample was chromatographed on a Superdex 200 10/300 GL column with a flow rate of 0.5 mL/min. Protein elution was followed by UV detection at 280 nm. Molecular weight standards are shown on top, and dashed line corresponds to the elution position of Rep protein alone.

Previous studies have indicated that Rep68/Rep78 has two regions that are required for oligomerization *in vitro*: A putative coiled-coiled region located in the OID and the AAA^+^ C-terminal domain [13]. We used size exclusion chromatography in order to determine if the individual domains are able to form higher molecular weight complexes in the presence of ssDNA. In the absence of ligands, Rep40 elutes as monomer with an estimated MW of 45 kDa (Figure 2A). Unlike Rep68, this profile does not change in the presence of ssDNA (Figure 2B and C). The OID alone also elutes as monomer (estimated MW ∼38 kDa; Figure 2D) and does not oligomerize in the presence of ssDNA (Figure 2E and F). Control experiments show that the OID is capable of forming a higher MW complex in the presence of RBS DNA (Figure 2G and H). By means of sedimentation velocity analyses on the interaction of OID with RBS, we determined that the 5∶1 OID∶RBS stoichiometry is formed at a salt concentration of 50 mM (data not shown). The conditions of the experiment shown in Figure 2G and H contain 200 mM NaCl, and support the formation of a complex with only 2 molecules of N208 bound to the RBS site (data not shown). Therefore, these results together with our observation that Rep68 oligomerizes in the presence of ssDNA, demonstrate a requirement of both domains to form the Rep68-ssDNA complex.

These studies further demonstrate that ssDNA elutes as a free form, suggesting that neither Rep40 nor the OID is interacting with the ssDNA under these conditions. This prompted us to examine the ssDNA binding affinities of the three protein constructs. Binding affinities were determined using fluorescent polarization on a fluorescein labeled poly-(dT)_38_ oligonucleotide. Figure S2 shows the binding isotherms for all three proteins, with a Rep40 binding constant of ∼3500 nM while OID binds ssDNA with higher affinity and a binding constant of ∼130 nM. As expected, Rep68 exhibits the highest affinity to ssDNA, with a binding constant of 23 nM. The large difference in affinities shown by the individual domains suggests a significant level of cooperativity involved during Rep68 binding to ssDNA.

**Figure 3:**
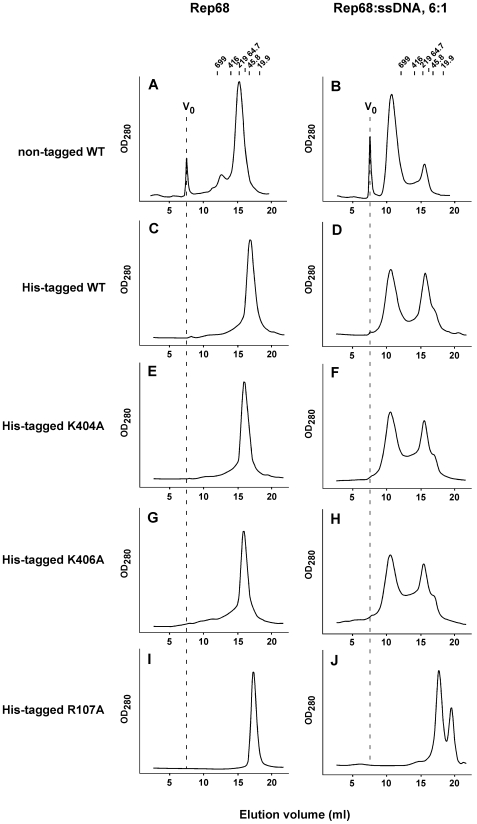
R107 is required for ssDNA-dependent Rep68. Rep proteins (16.6 µM) were incubated in the absence (A, C, E, G, I) or presence of 2.8 µM ssDNA (B, D, F, H, J). Fifty µL of sample was chromatographed on a Superose 6 10/300 GL column with a flow rate of 0.5 mL/min. Protein elution was followed by UV detection at 280 nm. Molecular weight standards are shown on top, and V_0_ position is represented as dashed line in Rep68 panels. (A and B) non-tagged Rep68; (C and D) His-Rep68 WT; (E and F) His-Rep68 K404A; (G and H) His-Rep68 K406A; and (I and J) His-Rep68 R107A.

This finding invited the question of whether residues involved in the respective DNA interactions by the individual domains influence the formation of the complex and thus contribute to the cooperativity. We have previously shown that B′ motif residues K404 and K406 located on β-hairpin-1 of the AAA^+^ domain of Rep40, are essential for ssDNA binding and helicase activity [16]. On the other hand, R107 located on the OID was shown to be essential for origin binding and nicking, as well as plasmid integration into the AAVS1 site [17]. It was later shown that this residue directly interacts with origin DNA [18]. His-tagged variants of all mutants were used and shown to elute as a single peaks in the absence of ssDNA (Figure 3C, E, G, and I). Albeit at somewhat lower efficiency, WT His-Rep68 oligomerizes in the presence of ssDNA (Figure 3D), and shows a similar elution profile as non-tagged Rep68 with ssDNA (Figure 3B). Mutation of either K404 or K406 did not affect His-Rep68 oligomerization *in vitro* (Figure 3F and H), indicating that these ssDNA binding residues of the helicase domain are not required for ssDNA-dependent oligomerization. In contrast, mutation of R107 residue completely eliminated His-Rep68 oligomerization, which was accompanied by the appearance of a new peak (ssDNA) at later elution volumes (Figure 3J). As a quality control and to rule out the possibility of an unfolded R107A mutant, we recorded the Circular Dichroism spectrum of both the HisRep68wt and R107 mutant proteins, which show similar profiles (Figure S3). Interestingly, R107A mutation is shifting Rep68 elution to species of lower molecular weight (Figure 3I), suggesting that this residue is involved in the Rep68 oligomeric interface directly or indirectly, in addition to its role in DNA binding.

Altogether, these findings suggest that R107, and by extension the OID, is critical for ssDNA-dependent Rep68 oligomerization *in vitro*.

### ssDNA promotes the formation of a ring-shaped double-octameric Rep68 complex

**Figure 4:**
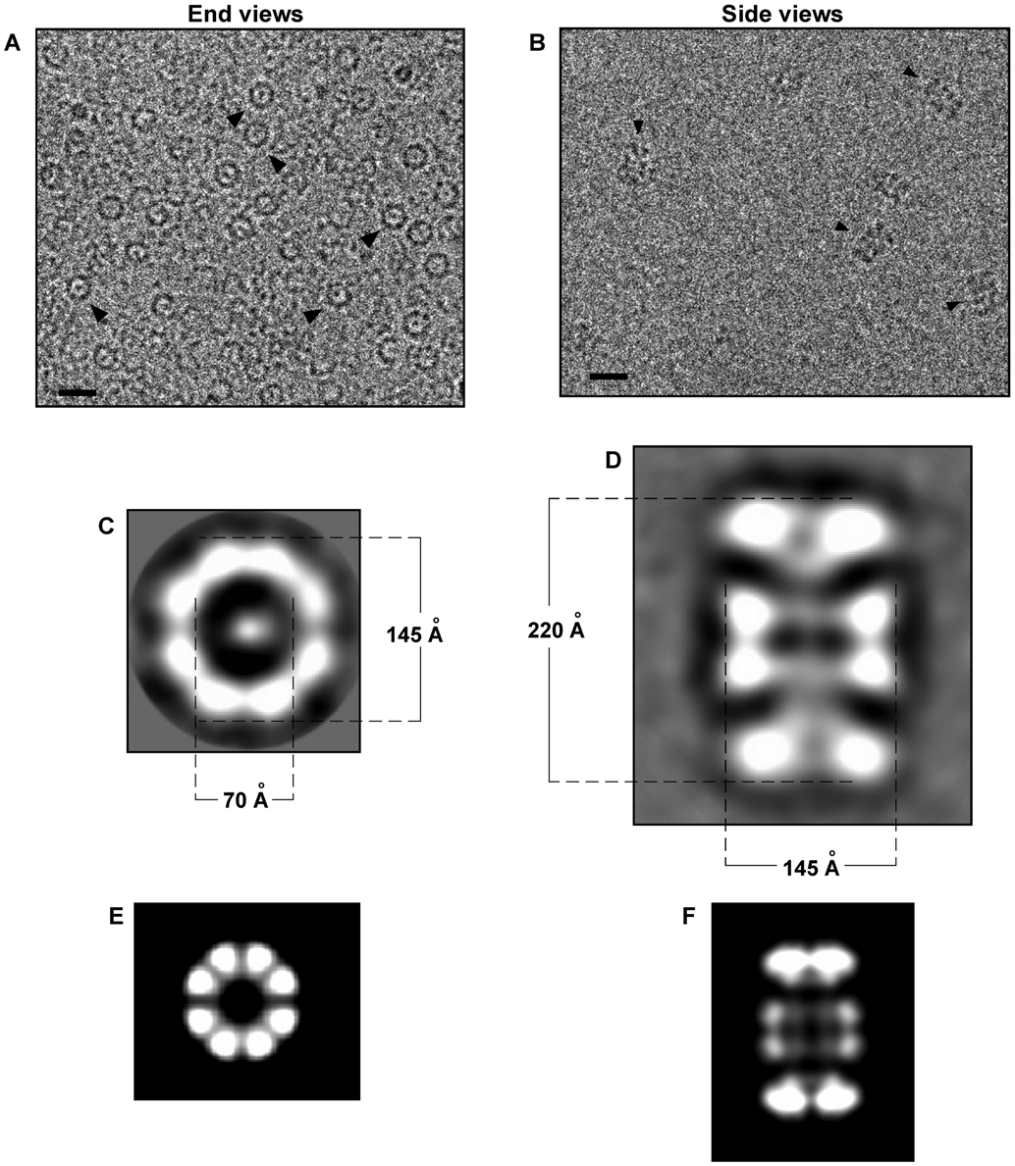
Cryo-electron microscopy of ssDNA-dependent oligomeric Rep68 rings. Rep68-ssDNa complex was purified by size-exclusion chromatography in buffer A, and central part of the peak was concentrated and used for further cryo-electron microscopy analysis. (A) Representative image of the ssDNA-Rep68 oligomer; ring-shaped end view are shown by arrowheads. Bar corresponds to 20 nm. (C) A representative class average of end views is shown; internal and external dimensions of the ring are shown in Angstroms. (B) The ssDNA-Rep68 oligomer was purified by size-exclusion chromatography in buffer A; central part of the peak was concentrated and mixed with n-octyl β-D-glucopyranoside just before cryo-EM analysis. Arrowheads indicate side views of the Rep68 oligomer. Bar corresponds to 20 nm. (D) A representative class of side views is shown. Dimensions in Angstrom are shown for the length and width of the oligomer. (E and F) Two-dimensional projections of a double-octameric Rep68; end view (E), and side view (F).

Our sedimentation velocity experiments suggest that Rep68 assembles into a ∼1 MDa complex in the presence of ssDNA. In order to gain structural information of the Rep68-ssDNA/Rep68 complex, cryo-electron microscopy (CEM) combined with single-particle analysis was used. For this, we purified the complex by size-exclusion chromatography and analyzed frozen samples by EM. We readily observed ring-shaped molecules (Figure 4A), a characteristic feature of AAA^+^ proteins, and SF3 helicases in particular [19], along with other views of the complex. Reference-free 2D alignment and classification of 852 rings was performed without imposing symmetry. Surprisingly, all classes showed a ring with eight-fold symmetry (a representative class is shown in Figure 4C). The octameric ring has an external diameter of 145 Å, and an internal diameter of 70 Å. In addition, we also observed elongated particles (Figure 4B). Using the same approach, 363 elongated particles were aligned without references and classified. A representative two-dimensional average view is shown in Figure 4D. The averaged view shows a clear two-fold symmetry, indicating that Rep68 assembles into double octameric rings in the presence of ssDNA. The dimensions of this double octamer are 145×220 Å. Interestingly, this analysis indicates that the two rings are assembled in opposite orientation. The overall shape of this side view strongly resembles the double-hexameric LTag [20], suggesting that Rep68 rings are interacting through their N-terminal domains. As a result, any additional domain attached to the N-terminal is likely to affect the formation of the complex. This possibility is in accordance with the observation that the His-tagged version of Rep68 does not form the complex as efficiently as the non-tagged protein (Figure 3C).

**Figure 5:**
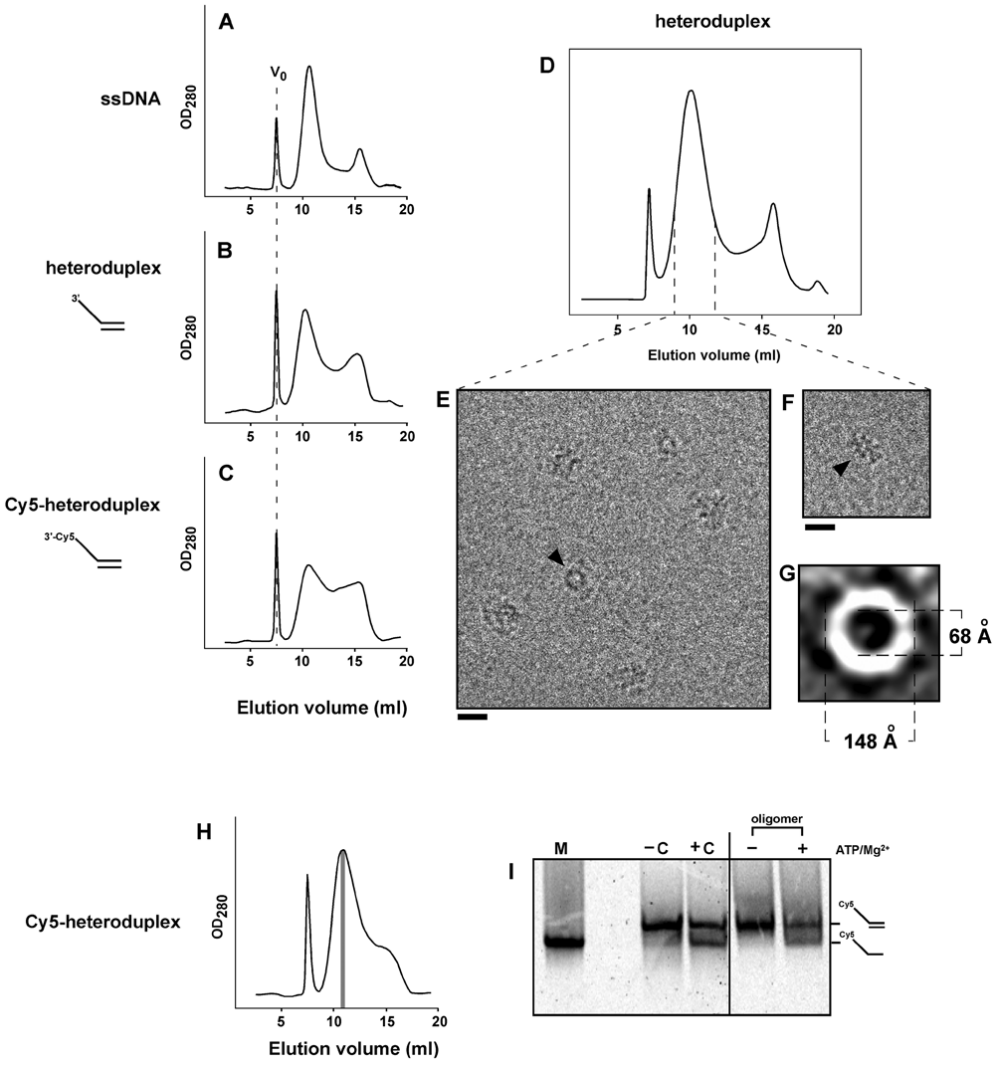
Rep68 forms an active helicase oligomer in the presence of ss-dsDNA heteroduplex. Rep68 (1 mg/ml, 16.6 µM) was incubated with 2.8 µM ssDNA (A), 2.8 µM heteroduplex (B), or 2.8 µM Cy5-heteroduplex (C) on ice for 30 min. After centrifugation, 50-µL of sample was chromatographed on a Superose 6 HR 10/30 column. Elution of Rep68 oligomers was followed by UV detection at 280 nm. V_0_ position is represented as dashed line in Rep68 panels. (D) Size-exclusion chromatography of Rep-heteroduplex in buffer A. Central part of the high-MW peak (dashed lines) was concentrated, and mixed with n-octyl β-D-glucopyranoside just before cryo-EM analysis. (E and F) Representative images of the heteroduplex-Rep68 oligomer; ring-shaped and elongated particles are shown by arrowheads. Bars correspond to 20 nm. (G) A representative class average of end views is shown; internal and external dimensions of the ring are shown in Angstroms. (H) Rep68 (16.6 µM) was incubated with 2.1 µM Cy5-heteroduplex, and chromatographed as above. Three hundred-µL fractions were collected, and fraction corresponding to the central part of the Cy5-hetroduplex/Rep68 oligomer (in gray) was 4-fold concentrated. (I) Concentrated oligomer was incubated in the absence (−) or presence (+) of 1 mM MgCl_2_/1 mM ATP for 30 min at 37°C. Helicase reactions were analyzed on 16% polyacrylamide gels combined with Cy5 detection. −C and +C correspond to the negative (substrate alone) and positive (substrate plus Rep68) controls respectively, which were incubated in the presence of 1 mM MgCl_2_/1 mM ATP. M, Cy5-ssDNA marker (oligo JM-37). At right, positions for Cy5-heteroduplex and Cy5-ssDNA are indicated.

In order to put the experimental projections into a structural context, a double octameric atomic model was generated and 2D projections were deduced for comparison. The model was built from the coordinates of the available AAV5 OID_1–197_ and the AAV2 Rep40_224–490_ structures [14],[21]. In this Rep68 model, the RBS interacting residue R107 is facing the internal channel and it is in proximity to the helicase domain, as it is found for the origin interacting residues in the double hexameric SV40 LTag structure [20]. Using the programs pdb2mrc and project3D from the EMAN package [22], a 3D map and 2D projections were obtained, respectively, without enforced symmetry. As shown in Figures 5E and F, the calculated 2D projections of the double-octameric Rep68 model resembles those observed in the CEM analyses: first, the ring is octameric, and second, the two rings indeed interact through the origin interaction domains. Projections generated with a double-hexameric ring model did not resemble the properties of the experimental projections (data not shown). In addition, inversion of the rings so that the helicase domains interact with each other did not yield projections that resemble the experimental CEM sideviews (data not shown). Therefore, the CEM data supports the conclusion that ssDNA promotes the *in vitro* oligomerization of Rep68 into head-to-head double octameric rings.

### Heteroduplex DNA substrate promotes the formation of an active double-octameric Rep68 complex

In order to examine the functionality of the observed Rep structure, heteroduplex DNA substrates were used to evaluate Rep68 oligomerization *in vitro* as compared to ssDNA containing complexes. Since a non-specific blunt-ended dsDNA does not promote Rep68 oligomerization (Figure 1), a heteroduplex substrate containing a 25-nucleotide ssDNA 3′-(poly-dT) tail juxtaposed to non-specific dsDNA was tested. As expected this ssDNA-dsDNA heteroduplex supported the formation of a Rep68 oligomer with an elution profile similar to that obtained with ssDNA (Figure 5A and B). This Rep68-heteroduplex complex (Figure 5D) was further purified and analyzed by CEM, revealing ring-shaped and elongated particles with dimensions that are similar to those obtained with ssDNA (Figure 5E and F). Reference-free 2D alignment of ring-shaped particles shows that Rep68 assembles into rings with dimensions of 148×68 Å, which are close to the dimensions of the Rep68-ssDNA ring (Figure 5G), suggesting that the rings in the Rep68-heteroduplex complex are also octameric.

The same heteroduplex DNA (in this case labeled with Cy5) was then used to perform helicase assays. As expected, the Cy5-labeled heteroduplex supported Rep68 oligomerization, albeit with less efficiency, into a complex with similar elution time (Figure 5C). Decreasing the heteroduplex concentration allowed efficient formation of the Rep68 oligomer and its subsequent purification (Figure 5H). Notably, in the presence, but not in the absence of ATP and magnesium, we observed that the purified Rep68-heteroduplex complex was indeed capable of unwinding DNA (Figure 5I), suggesting that Rep68 interacts with the ssDNA tail of the heteroduplex substrate, leading to the formation of an active helicase complex. Although we cannot discard the possibility that cofactors like ATP and Mg^2+^ could influence the ssDNA-dependent Rep68 oligomerization, we have observed that ATP, ATP/Mg^2+^, and ATP/Ca^2+^ -at concentrations used in our helicase assay- supported the formation of the same Rep68-ssDNA complex with the same efficiency when compared to ssDNA alone (Figure S4).

## Discussion

Superfamily 3 helicases include the viral initiator proteins BPV E1, SV40 LTag and AAV Rep68/78, among others. The function of these initiator proteins during viral DNA replication relies on their ability to oligomerize upon binding and subsequently melt their respective origin DNA. For E1 and LTag, it has been shown that they assemble into double hexameric rings on viral origin DNA, the oligomeric structure that is required for viral DNA unwinding during replication. Rep68/78 has also been shown to oligomerize in the presence of its origin DNA. Although it as been suggested that Rep68/78 forms hexamers, its oligomeric structure remains to be determined. However, based on the structural similarity of its AAA^+^ domain with E1 and Tag, it has been hypothesized that Rep68/78 would assemble into hexameric rings. Thus far no definitive experimental evidence has been presented that proves this hypothesis.

As these viral initiator proteins are necessarily multifunctional, we set out to investigate the ability of the DNA structure to modulate the oligomeric state of Rep68. We found that Rep68 forms a complex with RBS dsDNA containing five subunits of Rep68. This Rep68_5_-RBS complex is in accord with the crystal structure of the OID-RBS complex, which shows five OIDs bound to the RBS DNA [18]. Thus we have demonstrated that the AAA^+^ motor domain does not influence the number of Rep68 subunits that bind the RBS. However, the motor domain might influence the overall structure of both the OIDs and RBS DNA in the complex; therefore, additional structural investigation of the Rep68-RBS complex is necessary to elucidate this question.

Our results appear to be in contradiction with previous attempts to determine the stoichiometry of the Rep68/Rep78-origin complex but a closer look at the literature shows that this is not the case. For instance a report by Smith et al. [13] found that Rep78 forms a hexamer on an AAV ORI DNA molecule using gel filtration analysis. The DNA used is their study is 63 bp long, while we used a 26 bp DNA containing only the minimal RBS sequence. The chance of more Rep78 molecules binding to the longer DNA site is very likely. Moreover, the estimated stokes radius of the complex (64 Å) appears to be too small, particularly when the length of the DNA used is almost 215 Å. In the same report cross-linked Rep78 to AAV ORI DNA was analyzed on SDS-PAGE. The gel shows six clear bands, however, the presence of higher molecular weight complexes that did not enter the gel was not taken into account [13]. In another report, Muzyczka and colleagues introduced the concept that Rep68 can adopt different oligomeric states on ITR DNA, depending on Rep68 concentration as well as on the presence of ATP [12]. These investigators used native polyacrylamide gels to determine the molecular weight of the different Rep68:ITR complexes. However no precise determination of the Rep:DNA stoichiometry could be obtained. Nevertheless, as the authors point out, the AAV ITR DNA used contained additional contact points that are recognized by Rep68/78 [18],[23],[24], which could contribute to the binding of additional Rep68 molecules. Interestingly, at high Rep68 concentrations and in the presence of ATP, Rep68 binds the ITR mainly as a complex described as PDC5, which appears to contain 5 molecules of Rep68 [12]. Dignam et al. calculated a S_20,w_ value for the Rep68-RBS complex of 13.15 [11]. We obtained an S_20,w_ value of 11.5S. However, the difference of almost 2S indicates a real distinction between the two complexes. This disparity can be attributed to either a difference in the DNA substrate and/or buffer conditions. The RBS DNA site used by Digman (A stem) contains compatible overhangs of 4 and 6 nucleotides that could hybridize to produce longer DNA substrates where more Rep68 molecules could bind. In contrast, our RBS substrate has blunt ends. The claim by the authors that the sedimentation coefficient of 13.15 is “consisted with a tight complex comprised of two A-stem per six Rep68 subunits” supports the possibility of two concatenated A-stem DNA sites. In fact, a calculated sedimentation coefficient from the atomic model of the RBS site using the program HYDROPRO [25], predicts a sedimentation coefficient of ∼2.2S which is consistent with the experimental sedimentation coefficient of 2.4S that we obtained for the RBS site (data not shown). In contrast, Dignam et al. obtained a sedimentation coefficient for the RBS site of 3.28S. Moreover, their experimental conditions at low salt (50 and 100 mM) increases the likelihood of more Rep68 molecules binding. We propose that the pentameric assembly of Rep68 on the minimal RBS site could represent an intermediate complex that would require further assembly of Rep68 molecules in the presence of the full-length ITR origin molecule. Indeed, studies by Hickman et al. describe that they could detect a sixth OBD molecule using a longer RBS site than the 26-mer used in the crystallographic studies. However, using the same biochemical assay and full length Rep68, the number of molecules bound is now 7.5 [18]. Clearly, further biophysical and structural analysis of the Rep68-ITR complex in a purified form will provide a better understanding of the oligomeric nature of Rep68 when bound to the AAV ITR.

We further show that Rep68 self-assembles into double octamers upon binding ssDNA as well as heteroduplex helicase substrates, demonstrating a novel oligomeric structure of an SF3 helicase. This is in contrast to the hexameric-ring complexes formed by the equivalent AAA^+^ domains of both E1 and LTag upon binding ssDNA [10],[26]. Although our current structural models do not provide conclusive data indicating a molecular basis for the formation of octameric rings, it is likely that subunit-subunit interactions within the AAA^+^ ring are more stable in the octamer as compared to a possible hexamer conformation of Rep68 under the conditions used in our experiments. In addition, the ssDNA substrate might direct Rep68 into a conformation that matches the dimensions required to efficiently support both DNA replication and integration through a complex that is assembled from cellular replication factors.

We demonstrate that both the OID and the motor domain function cooperatively to assemble a double octamer and confer higher affinity binding to DNA. Therefore, the OID plays an important role in determining the symmetry of the ring by establishing subunit-subunit interactions in the OID ring that influence the interactions in the AAA^+^ ring. Although we cannot exclude the possibility that essential cofactors such ATP and magnesium could potentially influence the oligomeric state of Rep68, our results show that in presence of ssDNA this is not the case. Moreover, we have observed that ATP alone supports the formation of ring-shaped Rep68, whose dimensions are very similar to the rings obtained with ssDNA (data not shown). Preliminary gel filtration analyses suggest that this Rep68-ATP complex corresponds to a single Rep68 ring, and it requires both the OID and the AAA^+^ domains (data not shown). Altogether, our results suggest that Rep68 is poised to form octameric rather than hexameric rings. Nevertheless, it is noteworthy that the double octameric structure may only assemble under our experimental conditions. A detailed study of Rep68 in complex with ITR during the different steps of the terminal resolution reaction will be needed, in order to determine the biological relevance of this complex.

To date, the molecular mechanisms of Rep68/78 assembly during ITR resolution and the function(s) associated to each Rep oligomer are yet to be determined. Based on our observations, we hypothesized that DNA structure plays an important role in controlling the oligomeric nature of Rep68/78. In our *in vitro* conditions, we observe a stable Rep68:RBS complex that contains 5 molecules of Rep, and it may represents an initial complex that would require further assembly to initiate the ITR resolution reaction. It has been proposed that RBS melting is needed for the formation of hairpinned terminal resolution site (TRS), which is followed by nicking of the TRS by Rep68 [27]. Although there is no structural information of the Rep68/78 complex after RBS melting, it is likely that a ring-shaped Rep68/78 will form because of the ssDNA RBS that appears during melting as is the case with SV40 Tag and BPV E1.

Our results further show that Rep68 is functional as an octameric helicase, and we propose that both helicase rings may be active in this bidirectional complex. Although the proposed structure might have implications for our current replication model, the exact role of a double-octameric Rep68 in AAV DNA replication and/or site-specific integration remains to be determined. However, several scenarios are plausible. The current model for AAV DNA replication does not envision bidirectional replication [28], as it has been proposed for the SV40 and papilloma viruses. These viruses have a double-stranded DNA origin that contains two inverted repeats that are both recognized by the respective initiator protein. In contrast, AAV contains a single repeat (the RBS) in each ITR. Using LTag and E1 provide as precedence, Rep68 would be expected to require two inverted repeats in order to assemble a double octamer. In view of biochemical evidence, which suggests that Rep68 can form ternary complexes with 2 AAV ITRs [12], the Rep68 double octamer may coordinate the resolution of two ITR molecules (as may be the case of intermolecular unwinding). Another interesting scenario is the requirement of a double octamer during the refolding of ITR structures after completion of the ITR resolution and its subsequent duplication. Interestingly, two inverted RBS sequences are obtained after these steps, and, in theory, Rep68/78 proteins have the potential to recognize them and initiate their melting, followed by the formation of a double octamer, which would not only allow the refolding of the ITR structures but also the unwinding of the AAV dsDNA required for the following rounds of replication. Identifying the exact role of the Rep68 double octamer during AAV life cycle as well as its structural characterization will help to understand how Rep68 functions during the unwinding reaction.

In addition to the complexes presented here, it is plausible that Rep68 will assemble into additional different structure with other DNA substrates. Among the SF3 helicases, AAV Rep68/78 initiator protein is unique because of its ability to nick its origin DNA. During ITR resolution, the terminal resolution site (TRS) hairpin DNA is formed after RBS melting. This TRS hairpin is recognized and nicked by Rep68/78 in a sequence-specific manner. Therefore, we suggest that there exists a coordinated Rep68/78 oligomerization during origin DNA binding, melting, and nicking. Finally, the initiator protein Rep68/78 is also required for the site-specific integration of the viral DNA into the AAVS1 locus [1]. This locus contains RBS- and TRS-like sequences, which represent the minimal *cis* elements required for AAV integration [29]. Besides the recognition and nicking of these sequences [17],[30], Rep68/78 has been shown to form ternary complexes with AAV ITR and AAVS1 RBS DNAs, implying the interaction of two origins complexed through oligomeric Reps in this process [31].

Our findings demonstrate the versatility of Rep68 regarding its ability to assemble into different quaternary structures depending on the DNA substrate provided. Moreover, the data supports the idea that Rep68 can oligomerize through distinct pathways, with a pathway that relies on the cooperativity between the OID and the motor domain – as in the case of ssDNA - and a pathway that only requires the OID – in the case when RBS DNA is recognized. We propose that this flexibility in oligomerization provides Rep68 with the possibility to accommodate the different DNA structures it encounters during its involvement in all aspects of the AAV life cycle. Furthermore, our findings show a striking difference in oligomerization potential between Rep40 and Rep68, despite the fact that these two share the identical helicase domain. We hypothesize that AAV might have evolved to utilize a helicase domain that could support two different modes of DNA unwinding. This difference in the oligomerization-based mechanism may support the differential roles of Rep40 versus Rep68 in AAV DNA packaging and DNA replication/integration, respectively.

In conclusion, our study demonstrates that DNA structure modulates Rep68 oligomerization, requiring specific domain contribution of Rep68 depending on the DNA ligand. AAV ITR resolution and genome integration into the AAVS1 locus are complex reactions, where distinct Rep68-DNA complexes are expected to arise. Structural studies of these complexes are central for the elucidation of the molecular mechanisms of AAV DNA replication and site-specific integration into the human genome.

## Materials and Methods

### Protein expression and purification

Rep40 and OID-N208 Reps were expressed and purified as described [16],[32], except that the final buffer corresponded to Buffer A (25 mM Tris-HCl [pH 8.0 at 4°C], 200 mM NaCl, 5% glycerol, and 1 mM TCEP). His_6_-PreScission Protease (PP) cleavage site-Rep68 fusion protein was expressed in BL21(DE3)pLysS bacteria at 37°C for 3 h, in LB medium containing 1 mM IPTG. Cell pellets were lysed in 1∶1 Ni-Buffer A (20 mM Tris-HCl [pH 7.9 at 4°C], 500 mM NaCl, 10% glycerol, 0.05% NP-40, and 5 mM imidazole): B-PER solution (Pierce) containing protease inhibitors (2 µg/ml aprotinin, 2 µg/ml leupeptin, 1 µg/ml pepstatin A, and 600 µM PMSF). After five 10-s cycles of sonication, the fusion protein was purified using a Ni-column –equilibrated in Ni-buffer A. Protein eluted with 300 mM imidazole was desalted using PP buffer (50 mM Tris-HCl [pH 7.0 at 4°C], 200 mM NaCl, and 1 mM EDTA) and a HiPrep™ 26/10 desalting column (GE Healthcare). DTT was added to a final concentration of 1 mM, and His-PP tag was removed by PreScission protease treatment using 20 µg PP /mg His-PP-Rep68. After overnight incubation at 4°C, buffer was exchanged using the same desalting column and Ni-Buffer A. Subsequent Ni-column chromatography was performed to remove the uncleaved fusion protein, and untagged Rep68 was eluted with 50 mM imidazole. Rep68 (GE Healthcare) was finally purified by gel filtration chromatography using a HiLoad Superdex 200 16/60 column and Buffer A. Purified Rep68 was concentrated up to 20 µM (1.2 mg/ml), flash-frozen in liquid N_2_, and kept at −80°C until use. N-terminus His_6_-tagged WT and mutant Rep68 proteins were expressed and purified as above, except that proteins were directly concentrated after affinity purification, and loaded on the HiLoad Superdex 200 column.

### Analytical gel filtration chromatography

Rep68 (16.6 µM) was incubated in the absence or presence of 16.6 µM ssDNA (polydT_25_), 16.6 µM RBS dsDNA (generated with oligos JM-2: 5′ GCCTCAGTGAGCGAGCGAGCGCGCAGAG, and JM-20 CTCTGCGCGCTCGCTCGCTCACTGAGGC) or 1.4 µM non-specific IRF3 dsDNA [33] for 30 min on ice. Samples (50 µL) were chromatographed on a Superose 6 10/300 GL column (GE Healthcare) with a flow rate of 0.5 ml/min. For fractionation of Rep40 and OID proteins, Superdex 200 10/300 GL column (GE Healthcare) was used. Buffer A was used for all chromatographic analyses. Protein elution was detected by UV at 280 nm. For experiments using heteroduplexes, oligos JM-38 (5′-GGGAGAAGTGAAAGTGGGAA(T)_25_ ) and JM-40 (5′-TTCCCACTTTCACTTCTCCC) were used to generate the non-labeled heteroduplex, and oligos JM-37 (same sequence as JM-38 with the Cy5 molecule at 3′ end) and JM-40 were used to make the Cy5-heteroduplex. Formation of heteroduplexes was checked by gel filtration chromatography using the Superdex 75 column; in both cases a single peak was observed. MW standards of known Stokes radii (GE Healthcare) were used to estimate the MW and Stokes radius of Rep complexes.

### Analytical ultracentrifugation

Sedimentation velocity experiments were carried out using a Beckman Optima XL-I analytical ultracentrifuge (Beckman Coulter Inc.) equipped with a four-position AN-60Ti rotor. Rep68 (1 mg/ml) was incubated with 2.8 µM ssDNA (polydT_25_) or RBS dsDNA in buffer A. Samples in aluminum double sector cells were centrifuged at 45,000 rpm at 20°C. Concentration profiles were recorded using UV absorption (280 nm & 260 nm) and interference scanning optics, and analyzed using the program Sedfit [34]. We used a continuous distribution c(s) Lamm equation model with other prior knowledge that in this case is the number of species with different diffusion coefficients. We calculated the partial specific volume of the complex using the following equation:
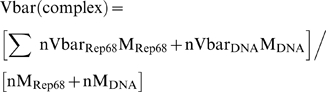



The vbar value used in the final calculation had a stoichiometry of 5∶1 (Rep68∶RBS), but other stoichiometries were also considered during the analysis. The addition of an extra molecule of Rep to the Vbar only increases its value by 0.0016 thus having a small effect on the final molecular weight but without affecting the final conclusions. The sedimentation coefficients were corrected to standard conditions (*S*_20,w_) using density and viscosity values calculated with SEDNTERP (http://www.rasmb.bbri.org/), a program developed by Hayes, Laue, and Philo.

For sedimentation equilibrium experiments, the Rep68-RBS complex was purified by gel filtration, and concentrated to an OD_260_ of 0.25 or 0.75. Each sample was analyzed at 4, 000, 5,000, and 7,000 rpm at 20°C. Radial scans of the absorbance at 260 and 280 nm were taken every 4 h, and equilibrium was determined by comparing successive scans using WinMatch, a program developed by Yphantis and colleagues (http://www.biotech.uconn.edu/auf/?i=aufftp). To obtain the background level at all three speeds, an over-speeding step at 42,000 rpm at 20°C for 6 h was performed, after which the speed was reduced to 4,000 rpm and radial scans were obtained. This procedure was repeated immediately for the other two speeds. After subtraction of the background level, the equilibrium concentration distributions were globally analyzed using HeteroAnalysis [35].

### Cryo-electron microscopy

Rep68-ssDNA samples were prepared by purification of the complex by size-exclusion chromatography (Superose 6 column). The central part of the peak was concentrated to about 0.4 mg/ml. For side view analysis, n-octyl β-D-glucopyranoside was added to a final concentration of 0.05%. This detergent did not affect ssDNA-dependent Rep68 oligomerization as determined by size-exclusion chromatography. Drops (3–4 µL) of sample were applied to glow-discharged Quantifoil EM 300-mesh grids with 2-µm holes, which were then blotted and plunged into a bath of liquid ethane (∼−180°C). Grids were analyzed in a Tecnai F20 transmission electron microscope, using the Tecnai low-dose package. Images of particles suspended in ice were collected at a microscope magnification of 50,000 and a defocus of 3 µm on a Tietz F415 CCD camera. Particles were selected using the boxer program from the EMAN software package [22]. Reference-free 2D alignment and classification were done with both the EMAN and SPIDER [36] software packages with similar results. For the Rep68-ssDNA/dsDNA complex, an identical approach was taken, except that a limited number of endviews was used for 2D classification.

### Protein modeling

The crystal structures of AAV5 OID_1–197_ [21] and AAV2 Rep40_225–490_ [14] were used to make a Rep68 atomic model, which lacks the linker region (residues 198–224) as well as the last 46 aminoacids. The orientation of the domains in the oligomeric rings was based in the known crystal structures of the E1 and LTag hexamers [37],[38]. The orientation of the rings in the double octamer was based on the CEM structure of the LTag double hexamer, in which both rings are interacting via their the N-terminal domains [20]. Dimensions of the double octamer were according to the cryo-EM data. 3D density maps at 30-Å resolution were obtained by using the EMAN program *pdb2mrc*. A series of 2D projections were obtained for each model by using the EMAN program *project3d* without symmetry imposed.

### Helicase activity

Control reaction (10 µl) contained 200 fmoles of Cy5-heteroduplex, 1.6 pmoles Rep68, 25 mM Tris pH 7.4 (25°C), 20 mM NaCl, 5% glycerol, 1 mM MgCl_2_, and 1 mM ATP. A negative control reaction contained the same components except Rep68. To test activity of the Cy5-heteroduplex/Rep68 complex, Rep68 (1 mg/ml; 16.6 µM) was incubated with 2.1 µM Cy5-heteroduplex in the presence of buffer A. After a 30-min incubation on ice, 100 µl of mix were loaded on the buffer A-equilibrated Superose 6 column. Fractions of 300 µl were collected, and the fraction corresponding to the central part of the complex peak was concentrated 4 times using Microcon concentrators (10 kDa cut-off; Millipore). The complex was incubated in the absence or presence of 1 mM MgCl_2_/1 mM ATP, with a final NaCl concentration of 20 mM. All reactions were carried out at 37°C for 30 min, and stopped by adding 7 µl of loading buffer (1× TBE, 0.5% SDS, 20% Glycerol), and immediately loaded on a native 16%-polyacrylamide gel. After electrophoresis, the Cy5-substrate and Cy5-ssDNA were detected using the STORM 860 phosphorimager set for red fluorescence detection. Oligo JM-37 was used as a marker for the ssDNA position.

### Gene/protein ID numbers

The DNA sequences of the proteins used in this manuscript are according to the AAV2 genome sequence.

AAV2 genome: GenBank accession number AF043303.

Rep68 protein: GenBank accession number AAC03774.

Rep40 protein: GenBank accession number AAC03776.

See Text S1 for supporting materials and methods.

## Supporting Information

Figure S1 Determination of Stoke's radius and radius of gyration for Rep68-RBS dsDNA and Rep68-ssDNA oligomers. (A) Kav vs logMW standard curve obtained with GE Healthcare markers (Thyroglobulin, Apoferritin, B-amylase, Alcohol dehydrogenase, Albumin, and Carbonic anhydrase). Positions of the Kav values for the Rep68-RBS, and Rep68-ssDNA complexes are shown. (B) Rs vs (−logKav)∧1/2 standard curve obtained with GE Healthcare markers Positions of the (−logKav)∧1/2 values for the Rep68-RBS, and Rep68-ssDNA complexes are shown. (C) Rep68-RBS and Rep68-ssDNA complexes were purified and concentrated as described in Materials and Methods, further analyzed by SAXS. The l(q) vs q(A^−1^) curves are shown for both complexes. Open circles: Rep68-RBS; closed circles: Rep68-ssDNA.
(0.21 MB TIF)

Figure S2 ssDNA binding affinities of Rep68, Origin binding domain (OBD), and Rep40 (helicase domain). Increasing concentrations of proteins were incubated with 5 nM of 5′6-carboxyfluorescein-labeled ssDNA as described. After incubation, the fluorescence anisotropy was measured using a fluorescence polarization system (Panvera). The fraction of DNA bound (B) vs protein concentration (nM) curves are shown; closed circle: Rep68; closed triangle: OID; and closed square: Rep40.
(0.13 MB TIF)

Figure S3 Far-UV CD spectra of His-Rep68WT and His-Rep68R107A proteins. Secondary structure of His-Rep68WT (solid line) and His-Rep68R107A (dashed line) at 0.2 µg/ml was monitored using CD spectroscopy. mdeg: millidegrees.
(0.07 MB TIF)

Figure S4 Effect of ATP, calcium, and magnesium on the ssDNA-dependent Rep68 oligomerization. Rep68 (16.6 µM) was incubated with a 25-mer ssDNA (2.8 µM) in the absence (A) or presence of 1 mM ATP (B), 1 mM ATP plus 1 mM CaCl_2_ (C), or 1 mM ATP plus 1 mM MgCl_2_ (D). Fifty-µl samples were chromatographed on a Superose 6 column, and fractions were analyzed for protein content. V_0_ position is represented as dashed line.
(0.09 MB TIF)

Text S1 Supporting materials and methods, and figure legends.
(0.05 MB DOC)
