# Supplementary figures and images for: DNA Structure Modulates the Oligomerization Properties of the AAV Initiator Protein Rep68
Source: PLoS Pathog. 2009 Jul 10;5(7):e1000513. doi: 10.1371/journal.ppat.1000513 (PMC2702170; doi:10.1371/journal.ppat.1000513)

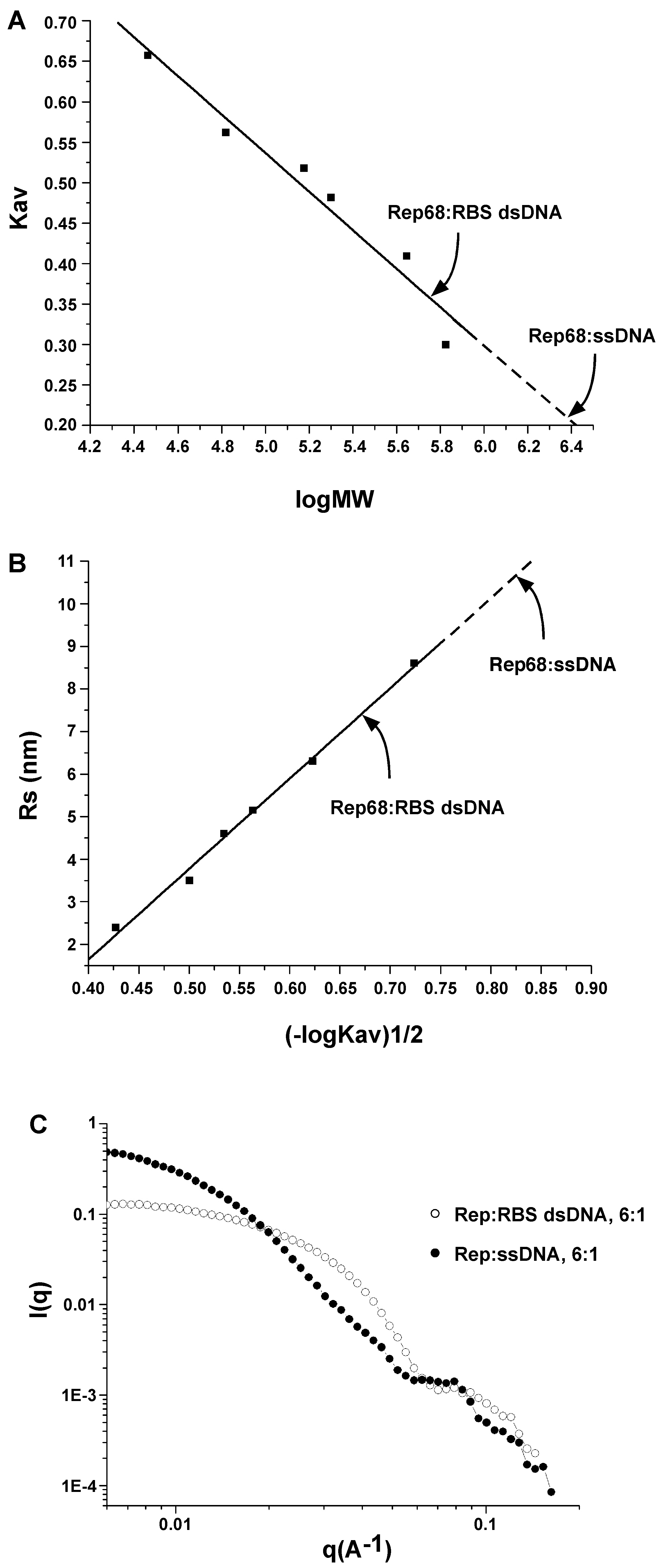

Supplement: Figure S1 — Determination of Stoke's radius and radius of gyration for Rep68-RBS dsDNA and Rep68-ssDNA oligomers. (A) Kav vs logMW standard curve obtained with GE Healthcare markers (Thyroglobulin, Apoferritin, B-amylase, Alcohol dehydrogenase, Albumin, and Carbonic anhydrase). Positions of the Kav values for the Rep68-RBS, and Rep68-ssDNA complexes are shown. (B) Rs vs (−logKav)∧1/2 standard curve obtained with GE Healthcare markers Positions of the (−logKav)∧1/2 values for the Rep68-RBS, and Rep68-ssDNA complexes are shown. (C) Rep68-RBS and Rep68-ssDNA complexes were purified and concentrated as described in Materials and Methods, further analyzed by SAXS. The l(q) vs q(A−1) curves are shown for both complexes. Open circles: Rep68-RBS; closed circles: Rep68-ssDNA. (0.21 MB TIF) [file ppat.1000513.s001.tif]

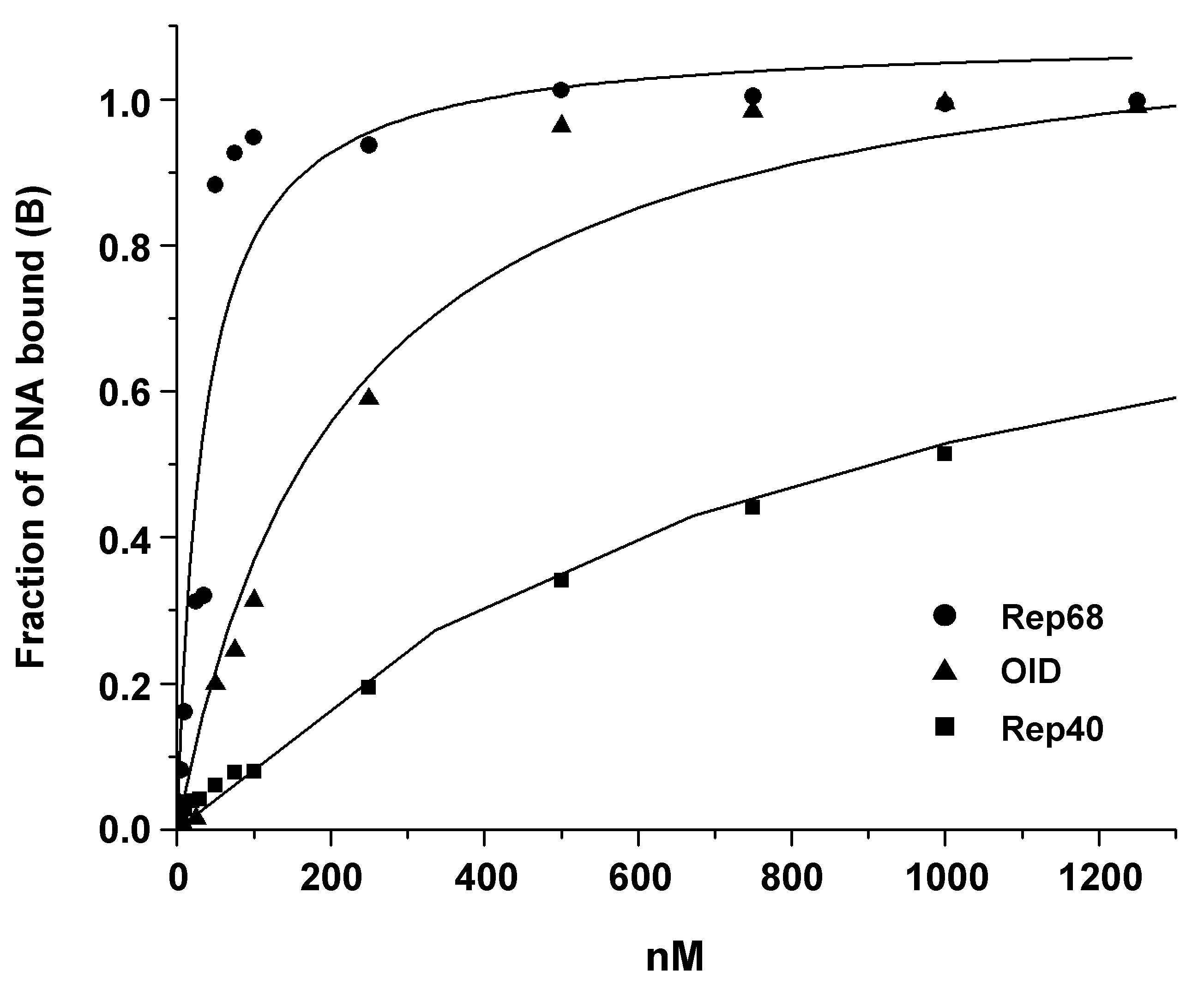

Supplement: Figure S2 — ssDNA binding affinities of Rep68, Origin binding domain (OBD), and Rep40 (helicase domain). Increasing concentrations of proteins were incubated with 5 nM of 5′6-carboxyfluorescein-labeled ssDNA as described. After incubation, the fluorescence anisotropy was measured using a fluorescence polarization system (Panvera). The fraction of DNA bound (B) vs protein concentration (nM) curves are shown; closed circle: Rep68; closed triangle: OID; and closed square: Rep40. (0.13 MB TIF) [file ppat.1000513.s002.tif]

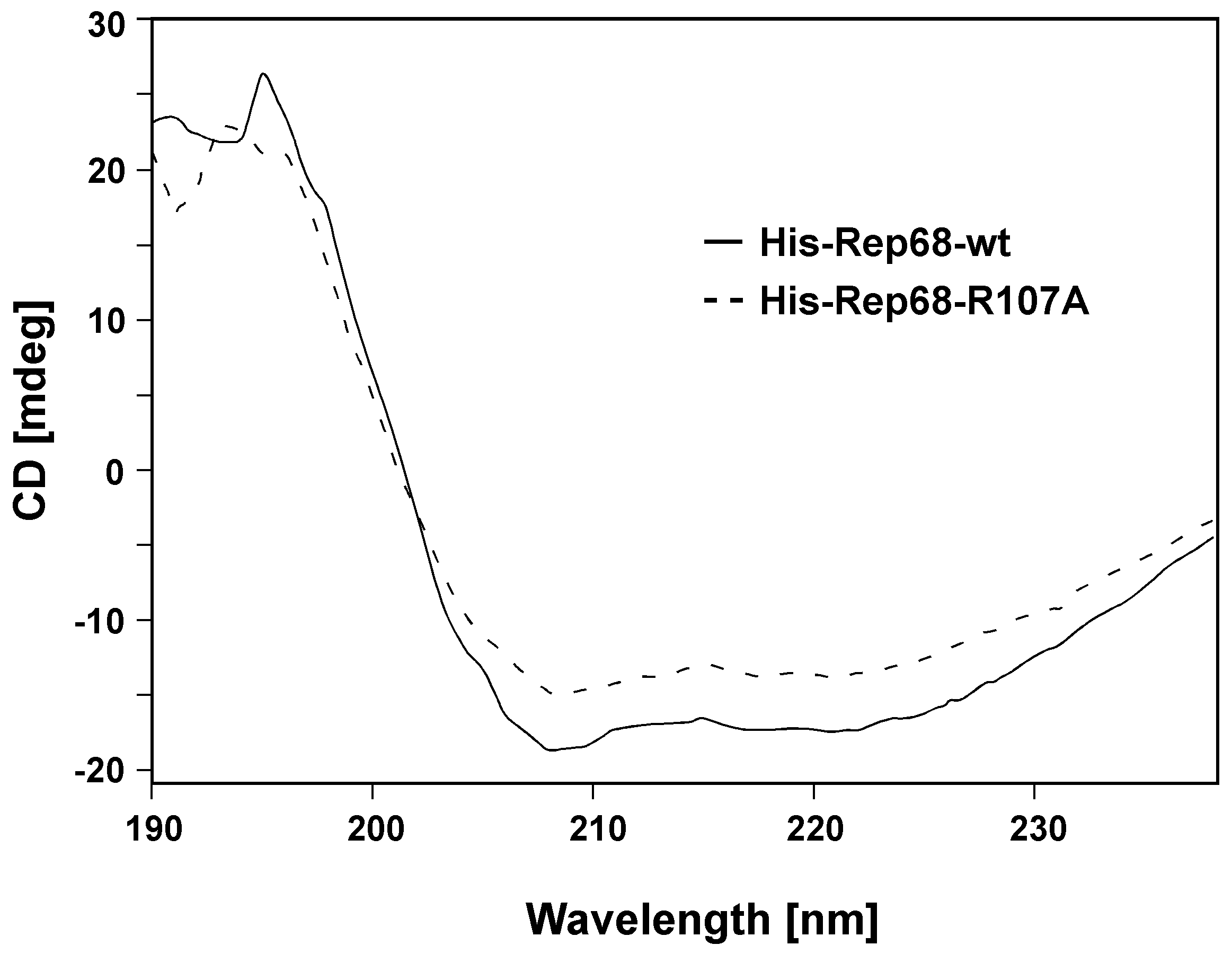

Supplement: Figure S3 — Far-UV CD spectra of His-Rep68WT and His-Rep68R107A proteins. Secondary structure of His-Rep68WT (solid line) and His-Rep68R107A (dashed line) at 0.2 µg/ml was monitored using CD spectroscopy. mdeg: millidegrees. (0.07 MB TIF) [file ppat.1000513.s003.tif]

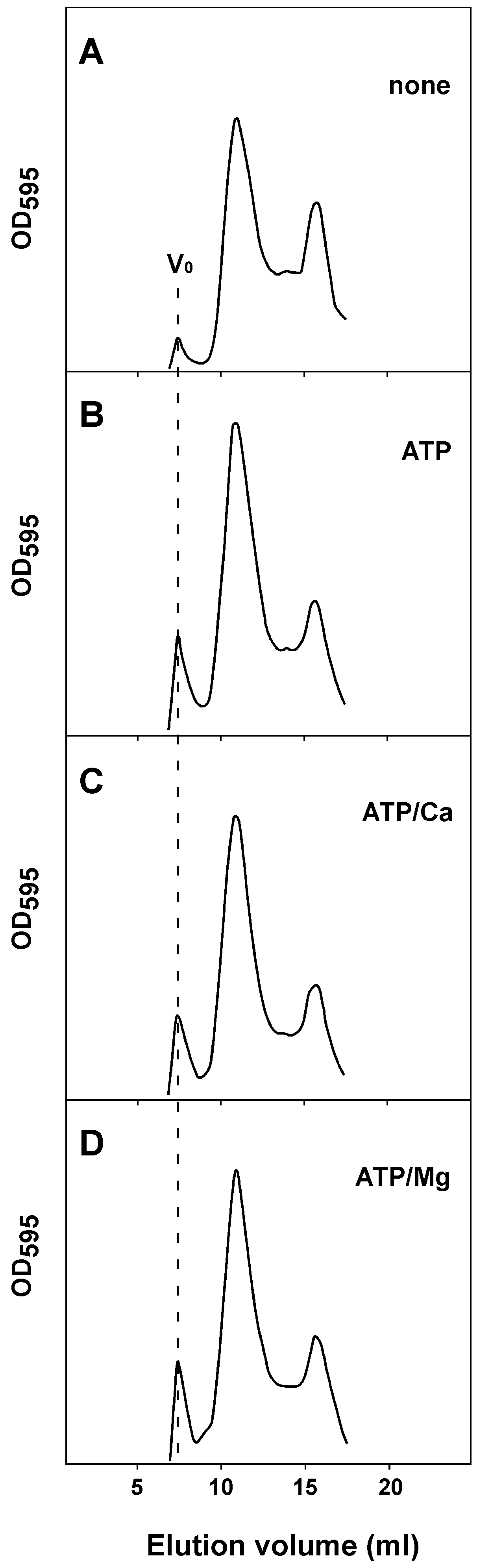

Supplement: Figure S4 — Effect of ATP, calcium, and magnesium on the ssDNA-dependent Rep68 oligomerization. Rep68 (16.6 µM) was incubated with a 25-mer ssDNA (2.8 µM) in the absence (A) or presence of 1 mM ATP (B), 1 mM ATP plus 1 mM CaCl2 (C), or 1 mM ATP plus 1 mM MgCl2 (D). Fifty-µl samples were chromatographed on a Superose 6 column, and fractions were analyzed for protein content. V0 position is represented as dashed line. (0.09 MB TIF) [file ppat.1000513.s004.tif]
