# Supplementary material for: DNA Structure Modulates the Oligomerization Properties of the AAV Initiator Protein Rep68
Source: PLoS Pathog. 2009 Jul 10;5(7):e1000513. doi: 10.1371/journal.ppat.1000513 (PMC2702170; doi:10.1371/journal.ppat.1000513)
Supplement: Text S1 — Supporting materials and methods, and figure legends. (0.05 MB DOC) [file ppat.1000513.s005.doc]

**Supplementary information**

**Materials and Methods**

**Small angle X-ray scattering (SAXS) data collection and processing**

SAXS measurements were carried out at undulator beamline 18-ID Biocat of the Advanced Photon Source, Argonne, IL, USA. The measurements employed a sample-detector distance of 2 m. Samples were purified on an FPLC using a Superose 6 10/300 GL column, concentrated to ~ 1mg/ml in buffer containing 25 mM Tris-HCl, 200mM NaCl, 1mM TCEP and 5% Glycerol. Samples were centrifuged for 15 minutes at 12,000 rpm immediately before data collection. The data was processed using standard procedures with program IgorPro with specific macros to process the data. The radius of gyration Rg was evaluated using the Guinier approximation with the program AutoRg [1]. The maximum dimension Dmax was computed with the program GNOM [2].

**Fluorescence Anisotropy Assa**y

Fluorescence anisotropy experiments were performed on a Beacon 2000 fluorescence polarization system (Panvera) as described [3]. The DNA substrate used was a single-stranded polydT_38_ oligo modified by a 5’6-carboxyfluorescein molecule (Integrated DNA Technologies). Binding assays were carried out at 20 °C for 15 min in a total volume of 100 µl in buffer containing 20 mM HEPES, pH 7.5, 50 mM NaCl, 5 nM DNA substrate, and protein. The fraction of DNA bound (*B*) was calculated using the following equation,

*B=([A]_X_ –[A]*_DNA_)/([*A]*_FINAL_ - [*A]*_DNA_) (see reference 3 for details)

**Analytical gel filtration chromatography**

Rep 68 (16.6 mM) and ssDNA (polydT_25_; 2.8 mM) were incubated in buffer A, in the absence or presence of 1 mM ATP, 1 mM ATP/1 mM MgCl_2_, or 1 mM ATP/1mM MgCl_2_ for 30 min on ice. Samples (50 mL) were chromatographed on a Superose 6 10/300 GL column (GE Healthcare) with a flow rate of 0.5 ml/min. Fractions (500 mL) were collected and analyzed for protein content using the Better Bradford Assay Kit (Pierce).

**Circular Dichroism (CD) spectroscopy**

His-Rep68WT and His-Rep68R107A were equilibrated in 25 mM Tris-H_2_SO_4_ (pH 8.0 at 4 °C), 150 mM NaF using Bio-Rad Bio-spin 6 columns. CD spectroscopy was performed on a Jasco J-810 spectropolarimeter. Protein samples (0.2 µg/ml) were analyzed at room temperature using a 1-mm pathlength cell, and the spectra were recorded in a wavelength range from 190-240 nm with 1-nm resolution. The scanning was done at 20 nm/min with a 4-s response time.

**References**

1. Konarev PV, Petoukhov MV, Volkov VV, Svergun DI (2006) ATSAS 2.1, a program package for small-angle scattering data analysis. J Appl Crystallogr 39: 277–286.

2. Svergun DI (1992) Determination of the regularization parameter in indirect transform methods uing perceptual criteria. J Appl Crystallogr 25: 495-503.

3. Yoon-Robarts M, Blouin AG, Bleker S, Kleinschmidt JA, Aggarwal AK, et al. (2004) Residues within the B' motif are critical for DNA binding by the superfamily 3 helicase Rep40 of adeno-associated virus type 2. J Biol Chem 279: 50472-50481.

**Figure legends**

**Figure S1. Determination of Stoke’s radius and radius of gyration for Rep68-RBS dsDNA and Rep68-ssDNA oligomers.** (A) Kav vs logMW standard curve obtained with GE Healthcare markers (Thyroglobulin, Apoferritin, B-amylase, Alcohol dehydrogenase, Albumin, and Carbonic anhydrase). Positions of the Kav values for the Rep68-RBS, and Rep68-ssDNA complexes are shown. (B) Rs vs (–logKav)^1/2 standard curve obtained with GE Healthcare markers Positions of the (–logKav)^1/2 values for the Rep68-RBS, and Rep68-ssDNA complexes are shown. (C) Rep68-RBS and Rep68-ssDNA complexes were purified and concentrated as described in Materials and Methods, further analyzed by SAXS. The l(q) vs q(A^-1^) curves are shown for both complexes. Open circles: Rep68-RBS; closed circles: Rep68-ssDNA.

**Figure S2. ssDNA binding affinities of Rep68, Origin binding domain (OBD), and Rep40 (helicase domain).** Increasing concentrations of proteins were incubated with 5 nM of 5’6-carboxyfluorescein-labeled ssDNA as described. After incubation, the fluorescence anisotropy was measured using a fluorescence polarization system (Panvera). The fraction of DNA bound (B) vs protein concentration (nM) curves are shown; closed circle: Rep68; closed triangle: OID; and closed square: Rep40.

**Figure S3. Far-UV CD spectra of His-Rep68WT and His-Rep68R107A proteins.** Secondary structure of His-Rep68WT (solid line) and His-Rep68R107A (dashed line) at 0.2 µg/ml was monitored using CD spectroscopy. mdeg : millidegrees.

**Figure S4. Effect of ATP, calcium, and magnesium on the ssDNA-dependent Rep68 oligomerization.** Rep68 (16.6 µM) was incubated with a 25-mer ssDNA (2.8 µM) in the absence (A) or presence of 1 mM ATP (B), 1 mM ATP plus 1 mM CaCl_2_ (C), or 1 mM ATP plus 1 mM MgCl_2_ (D). Fifty-µl samples were chromatographed on a Superose 6 column, and fractions were analyzed for protein content. V_0_ position is represented as dashed line.
